# Xanthine oxidase inhibitors in ischaemic heart disease

**DOI:** 10.5830/CVJA-2016-068

**Published:** 2017

**Authors:** Mihnea Zdrenghea, Adela Sitar-Tǎut,, Gabriel Cismaru, Dumitru Zdrenghea, Dana Pop

**Affiliations:** Department of Haematology, Iuliu Hațieganu University of Medicine and Pharmacy, Cluj-Napoca, Romania; Department of Cardiology, Iuliu Hațieganu University of Medicine and Pharmacy, Cluj-Napoca, Romania; Department of Cardiology, Iuliu Hațieganu University of Medicine and Pharmacy, Cluj-Napoca, Romania; Department of Cardiology, Iuliu Hațieganu University of Medicine and Pharmacy, Cluj-Napoca, Romania; Department of Cardiology, Iuliu Hațieganu University of Medicine and Pharmacy, Cluj-Napoca, Romania

**Keywords:** xanthine oxydase inhibitors, ischaemic heart disease, uric acid

## Abstract

Increased uric acid levels are correlated with cardiovascular disease, particularly with ischaemic heart disease. Xanthine oxidase inhibitors, especially allopurinol, lower the risk of ischaemic heart disease due to their effects on reactive oxygen species and endothelial function. In chronic stable angina pectoris, allopurinol increases the median time to ST depression, time to chest pain, and total exercise time. On the other hand, it has been reported that allopurinol has a beneficial effect on ischaemic patients referred for angioplasty, but there are insufficient data regarding its effect on acute myocardial infarction patients. Moreover, other important actions of allopurinol are regression of left ventricular hypertrophy and improvement in the results of cardiac rehabilitation. The efficacy of allopurinol has recently been acknowledged by the European Society of Cardiology guidelines for stable angina pectoris, but the particular role of allopurinol in ischaemic heart disease patients is not fully established.

## Introduction

There are many cardiovascular conditions and risk factors associated with elevated uric acid levels.^1^ Uric acid favours hypertriglyceridaemia, being involved in the increase of liver protein synthesis and turnover.^2^ Hyperuricaemia has been associated with an increased incidence and prevalence of hypertension, stroke and carotid, peripheral and coronary atherosclerotic vascular disease.^1^

There is also a correlation between elevated levels of uric acid and inflammatory markers, including C-reactive protein (CRP), plasminogen activator inhibitor type I, and soluble intercellular adhesion molecule (ICAM).^3^ All these factors represent another possible link between uric acid and atherosclerosis, especially ischaemic heart disease.^1^ In a review on uric acid levels in cadiovascular disease, Kanbay et al. cite eight prospective studies based on medical and post mortem records, and coronary events registries, which demonstrate an increased risk of coronary heart disease in subjects with hyperuricaemia, with odd ratios (OR) between 1.12 and 2.30.^4^

These data suggest that a decrease in serum uric acid level could be beneficial in patients either at risk for or with established ischaemic heart disease. The most commonly used drugs to decrease uric acid levels are inhibitors of xanthine oxidase (XO). This enzyme is involved in uric acid synthesis, in the production of superoxide radicals and, consequently, in atherosclerosis.^5^ Therefore, a decrease in its activity may have anti-atherogenic and anti-ischaemic effects.^6^ There are three clinically available XO inhibitors: allopurinol, oxypurinol and febuxostat, the first being most widely used in clinical practice.^7^

The many potential pharmacological cardiovascular benefits of XO inhibitors include improvement in endothelial function, decrease in tissue oxidative stress, increase in ATP synthesis in ischaemic tissue, and improvement in exercise-induced ischaemia. XO inhibitors may also be beneficial in prevention of primary cardiovascular disease, left ventricular hypertrophy, acute coronary syndrome, stroke and heart failure.^6^ We will briefly discuss the main areas in which XO inhibitors could be or have already proven useful.

## Anti-atherogenic effects

The anti-atherogenic effects of XO inhibitors have mainly been studied in relation to endothelial function and oxidative stress parameters.^8^ Inflammatory markers and lipid profile have also been considered.^9^ XO represents a source of reactive oxygen species that results in both endothelial dysfunction and vascular inflammation. Consequently, lowering serum uric acid levels through XO inhibitors has anti-atherogenic effects.

A review and meta-analysis of 40 studies reports that circulating markers of oxidative stress, such as malonaldehyde, were significantly decreased by XO inhibitors in six of the studies.^9^ Other studies found that brachial artery flow-mediated dilatation was increased, with an OR of 2.50. The forearm blood flow response to acetylcholine infusion was increased by 60.68%.^9^

In their 2013 review, Kanbay et al. analysed the relationship between reduction in uric acid level and improvement of endothelial dysfunction in patients with diseases including congestive heart failure, the metabolic syndrome, diabetes and chronic kidney disease. In all cases, the improvement was significant, between 25 and 100%.^4^ On the other hand, studies on inflammation and lipid profile yielded controversial data. XO inhibitors did not influence CRP levels in several studies, CRP being decreased in only one.^10^ ICAM was also reduced in only one of the studies.^9^ Fibrinogen, interleukin 6 (IL-6), vascular endothelial growth factors (VEGF) and E-selectin levels were not affected. 9 One study found an improved lipid profile, but two further studies did not.^8^

Ziga et al. reported that in 40 hyperuricaemic patients, levels of triglycerides, total cholesterol, low-density lipoprotein (LDL) cholesterol and high-density lipoprotein (HDL) cholesterol were slightly increased after three months of allopurinol treatment.^11^ The authors suggested that in patients with the metabolic syndrome, lipid profile should be monitored after the initiation of allopurinol treatment, as the atherogenic index is increased in these patients. Renin and B-type natriuretic peptide (BNP) levels were decreased by XO inhibitors, this finding being of special relevance to ischaemic and heart failure patients.^9^,^12^

## Angina pectoris

The use of allopurinol improves chronic stable angina. Angina pectoris has a high prevalence of 5.7% in men and 6.7% in women, not all of them being referred for interventional cardiology. In these patients, allopurinol could be useful to increase exercise time, time to 1-mm ST depression, and time to angina.^13^ This is the main reason why allopurinol is recommended when classical anti-anginal drugs are contra-indicated or not efficient in controlling angina. The previous assertion is in agreement with the 2013 European Society of Cardiology guidelines on the management of stable coronary heart disease, which recommend 600 mg/day allopurinol under the ‘other drugs’ heading.^14^

Allopurinol has been found to improve exercise capacity by increasing ATP production for the same myocardial oxygen supply. Therefore allopurinol increases not only exercise capacity, but also the double product and peak effort.^15^ On the other hand, classical anti-anginal drugs increase exercise capacity, but not the double product or myocardial energy production.^15^

Other beneficial effects of allopurinol are related to endothelial function and coronary vasoconstriction. Rajendra et al. studied endothelial function assessed by forearm venous occlusion plethysmography, flow-mediated dilation and pulsewave plethysmography in 80 patients with coronary heart disease. Compared to the placebo group, allopurinol improved endothelium-dependent vasodilation and completely eliminated oxidative stress.^16^

In another study, Noman et al. investigated the effects of high-dose allopurinol on exercise in patients with stable angina pectoris.^17^ The study included 65 patients with positive stress testing, in whom 600 mg/day allopurinol increased time to ST depression from 232 to 298 seconds. The duration of exercise was consequently increased from 301 to 393 seconds. The time to chest pain was increased too, from 234 to 304 seconds, the difference being highly significant. On the contrary, the effect on the number of angina episodes/week or number of tablets of glyceryl trinitrate/week was not significant in comparison with placebo.

Unfortunately, there are further studies which do not confirm the aforementioned results. They describe no significant improvement of exercise capacity in patients with stable angina treated with allopurinol.^9^,^15^

Rekraj et al. studied the effect of high-dose allopurinol on left ventricular hypertrophy and endothelial function in patients with chronic stable angina. Using 600 mg allopurinol daily, flowmediated vasodilation increased by 0.82 ± 1.8% at nine months, from 4.1 ± 2.1% at baseline. On the other hand, in the placebo group, the initial flow-mediated vasodilation of 5.68% decreased by 0.69 ± 2.8% at nine months (p = 0.017).18 The data suggested the need for long-term treatment with allopurinol in order to obtain anti-atherosclerotic effects.

The same article showed a nine-month left ventricular mass (LVM) and left ventricular mass index (LVMI) decrease of 5.2 g and 2.2 g/m^2^, respectively, in the allopurinol group, versus 1.3 g and 0.53 g/m^2^ in the placebo group, the difference being highly significant. They also determined the augmentation index (AIx), which was lower in the allopurinol group, suggesting not only improvement in endothelial function, but also less vascular remodelling. The results suggest that allopurinol is useful in hypertensive patients with ischaemic heart disease.

Agarwal et al. recently presented a meta-analysis of 10 studies on the effects of allopurinol in 738 hypertensive patients.^19^ Compared to the control group, allopurinol-treated patients displayed a 3.3-mmHg systolic and a 1.3-mmHg diastolic blood pressure decrease.

## Other manifestations of ischaemic heart disease

There are many further studies on the effects of allopurinol in ischaemic heart disease but data are insufficient to allow the formulation of strong evidence-based guidelines.^20^ Beneficial effects were reported mainly in patients with myocardial revascularisation with coronary artery bypass surgery or angioplasty post myocardial infarction.

Available data allow advocating a role for allopurinol in decreasing the number of complications, including arrhythmias, and improving myocardial function in revascularised patients, sustained by experimental data on the effect of allopurinol on cardiomyocyte apoptosis in rats after myocardial infarction.^20^ Xiao et al. reported a decrease in apoptosis measured through caspase activity in non-infarcted myocardial areas in rats with infarction.^21^ The study suggests myocardial protection through allopurinol, not only in chronic forms of ischaemic heart disease, but also in acute coronary syndromes.>

In 2010, Rentoukas et al. reported that acute myocardial infarction patients submitted to primary percutaneous angioplasty and treated with a loading dose of 400 mg allopurinol followed by 100 mg daily for one month displayed a more effective ST-elevation recovery and lower peak values of troponin, CK-MB and creatine phosphokinase (CPK), along with a 13% decrease in major adverse cardiac effects at one-month follow up.^22^

In a recent review, Grimaldi-Bensouda et al. discussed the impact of allopurinol on the risk of myocardial infarction and compared the drug to colchicine. The myocardial infarction OR in the allopurinol group was 0.80, compared to 1.17 in the colchicine group.^23^

In a critical review, Robert et al. emphasised that the effects of allopurinol on cardiovascular disease are mediated by XO inhibitors via free radicals and inhibition of oxidative stress.^24^Gladden et al. studied the effect of allopurinol on systolic and diastolic left ventricular function in rats with volume overload from aorto-caval fistula.^25^ Allopurinol increased left ventricular (LV) contractility and ejection fraction, but did not alter LV dilation and diastolic pressure/wall stress rate as a measure of diastolic function, despite XO activity being increased in human myocytes with volume overload.^25^ The aforementioned data may explain why, in the EXACT-HF study in hyperurcaemic heart failure patients, allopurinol failed to improve LV ejection fraction, symptoms, exercise capacity and time to hospitalisation.^26^

Recently, Valbusa et al. reported an increase in incidence of atrial fibrillation in type 2 diabetes patients with hyperuricaemia (10.5% in 10 years) with an OR of 2.43, but there are insufficient data to confirm that this increase could be prevented by using XO inhibitors. To date, no trial has been conducted to examine the effect of allopurinol on atrial fibrillation.^27^

Beveridge et al. reported that allopurinol was associated with significant functional improvement in older rehabilitation patients, including those with cardiovascular disease.^28^ The improvement in functional status was demonstrated using the Barthel score, which was higher in the allopurinol group (4.7 vs 3.6; p = 0.002). These findings could be attributed to the increase in ATP production by allopurinol.^29^

Sanchis-Gomar analysed post-exercise cardiovascular markers of injury in 12 football players. They found that 300 mg allopurinol before a football game had no significant effect on these markers, except for promedulin levels, which were higher in the placebo than in the allopurinol group. 29

Many controversial issues will be answered through the CARES trial, which aims at studying the effects of allopurinol and febuxostat in patients with gout and cardiovascular co-morbidities.^30^ This study includes 7 500 patients with gout and cardiovascular disease, followed up for five years. Cardiovascular end-points, composite cardiovascular death, non-fatal myocardial infarction, non-fatal stroke, and unstable angina requiring urgent coronary revascularisation will be taken into consideration.

## Conclusion

XO inhibitors have proven efficacy as second-line drugs in patients with chronic stable ischaemic heart disease, and are recommended in this setting by current evidence-based guidelines. In other manifestations of ischaemic heart disease, data are controversial and further investigation is warranted.
